# The abrupt climate change near 4,400 yr BP on the cultural transition in Yuchisi, China and its global linkage

**DOI:** 10.1038/srep27723

**Published:** 2016-06-10

**Authors:** Jianjun Wang, Liguang Sun, Liqi Chen, Libin Xu, Yuhong Wang, Xinming Wang

**Affiliations:** 1 Key Laboratory of Global Change and Marine-Atmospheric Chemistry, Third Institute of Oceanography, State Oceanic Administration, Xiamen, China; 2 Institute of Polar Environment, University of Science and Technology of China, Hefei, China; 3 Zhangjiagang Water Supply & Drainage Company, Zhangjiagang, China; 4 National Institute of Health, USA; 5 Guangzhou Institute of Geochemistry, Chinese Academy of Sciences, Guangzhou, China

## Abstract

Extreme climatic events have profound impacts on human society. Here we present the results of a study of organic biomarkers within a sedimentary section at the archaeological site of Yuchisi, eastern China, in order to reconstruct climatic variability during the Dawenkou (5,050–4,400 yr BP) and Longshan (4,400–4,000 yr BP) cultures. At ~4,400 yr BP, within the cultural transition horizon, abrupt changes in biomarkers, such as the fatty acid ratio C_18:2_/C_18:0_, 2C_31_/(C_27_ + C_29_), *n*-C_18_-ol and *n*-C_30_-ol, indicate the occurrence of local climate changes over the course of a few decades. These changes occurred during the transition from the Holocene warm period to a subsequent cold period which lasted for the following 600 years. This climatic shift has been recorded at numerous sites worldwide, and it is likely to have been the main cause of the widespread collapse of many isolated cultures at that time. The palaeoclimatic and archaeological data from the Yuchisi sediments may provide new insights into the relationship between climate change and prehistoric cultural transitions.

Extreme climatic events can have a devastating impact on human societal and ecological systems^1^,^2^, and they may have played an important role in human history^3^,^4^,^5^,^6^,^7^,^8^. Major steps in the evolution of African hominids and other vertebrates at 2.8 Ma, 1.7 Ma, and 1.0 Ma coincided with climatic shifts to more arid and open conditions, suggesting that some Plio-Pleistocene speciation events may have been climatically mediated^9^. In southwest Asia, the climatic impact of the Younger Dryas (about 12,900 to 11,600 years before the present, BP) changed the developmental trajectory of the Natufians, who replaced seasonally nomadic hunting and gathering activities with new labor-intensive subsistence strategies of plant cultivation and animal husbandry^10^. The collapse of the Akkadian empire around 4,170 yr BP was probably related to the onset of arid conditions that are recorded in a sediment core from the Gulf of Oman^11^. The collapse of the classic Mayan Empire was connected to the abrupt shift to arid conditions in the Yucatan Peninsula between 1,300–1,100 calendar yr BP, as recorded by several lake sediment records^5^,^12^. Furthermore, during the past 2,000 years, episodes of social destabilization, wars, and dynastic cycles in China were more frequent during intervals of cold or drought^13^,^14^,^15^.

Most of the studies on the association between historic climate variability and human societal evolution are based on records from lake sediments, marine sediments, and ice cores, and are rarely obtained directly from archaeological sites^1^. Because the timing of climate and societal changes cannot always be determined with sufficient accuracy and precision, a time lag between climate changes and the societal or vegetation response is sometimes assumed^16^. Time lags make it more difficult to distinguish the climatic impacts on a civilization from other social and economic influences, and thus to understand the detailed mechanism of these impacts.

In this study, we obtained a paleoclimatic record from a 460-cm-long sediment record from the largest and most integrated archaeological site in Yuchisi, Mengcheng County, Anhui Province, eastern China (Fig. 1). Based on the archaeological evidence, the Yuchisi site was an important geographical location: a rudimentary city with flourishing architecture and an important Neolithic cultural centre that attracted a significant population during the Neolithic period^17^,^18^. As identified by the archaeological analysis, the Yuchisi site recorded two important Chinese prehistoric cultures: the Late Dawenkou Culture (5,050–4,400 yr BP) and the Longshan Culture (4,400–4,000 yr BP)^17^,^18^,^19^,^20^,^21^. The location of the studied sediment profile (YC1) was carefully selected in order to avoid possible disturbance by human activity. It was located in an open area which apparently had not been used for habitation, cultivation, or metalworking. It is the least disturbed site in the vicinity. The linear age-depth relationship confirms that no significant human disturbance occurred at the location^21^. A previous study of the relationship between the pollen record and Asian summer monsoon variations also suggest that the sedimentary record of YC1 was mainly affected by natural climate variations^21^. Previous analyses of pollen assemblages and element concentrations in the sediment profile also revealed an episode of unusually dry climate occurred at 4,400 yr BP in the region^21^. In the present study, we analyzed organic biomarkers in the sediments and use the results to consider how the near-simultaneous climate changes and cultural transition at the Yuchisi site are reflected in similar occurrences on a global basis.

## Results

The down-section variations in TOC, Chenopodiaceae/total pollen ratio, and records of typical biomarkers are illustrated in Fig. 2. Within the profile, the carbon number of alkanes ranges from 15 to 33, with odd-to-even predominance. The carbon number of alkanols ranges from 14 to 34, with even-to-odd predominance; peaks of the C_18_ *n*-alkanols dominate the alcohol fractions. The carbon number of fatty acids ranges from 14 to 24, with even-to-odd preference; and the main fractions of the fatty acids are C_16_ and C_18_.

The interval of 460–450 cm is the original loess layer and is uninfluenced by human activity. Above 450 cm, the indexes of typical biomarkers (fatty acid ratio C_18:2_/C_18:0_, alkane ratio 2C_31_/(C_27_ + C_29_), and levels of *n*-C_18_-ol and *n*-C_30_-ol) exhibit a number of distinct oscillations (Fig. 2). The first significant change in TOC and other biomarkers (including the fatty acid ratio C_18:2_/C_18:0_, alkane ratio 2C_31_/(C_27_ + C_29_), *n*-C_18_-ol and *n*-C_30_-ol) occurs at the depth of 450 cm, dated to 5,050 yr BP. The datum of 5,050 yr BP corresponds to the initial settlement of the area by the Dawenkou Culture and to the first changes in the biomarkers, especially in TOC and *n*-C_30_-ol.

The second major change in the biomarkers occurs at the levels dated to ~4,400 yr BP, at depths of 212–214, 208–210 and 198–200 cm, and is consistent with the transition from the Dawenkou Culture to the Longshan Culture. The fatty acid C_18:2_/C_18:0_ ratio decreases rapidly from 0.12 to 0.06 between 220 cm and 200 cm, then increases to 0.22 at 200 cm, and decreases to 0.07 at 196 cm (Fig. 2). Unsaturated fatty acids may enhance the ability of organisms to survive in a cold climate by keeping cell membranes stable and flexible^22^,^23^. Although it has been suggested that the acid is potentially vulnerable to degradation, the ratio of sedimentary *n*-C_18:2_ and *n*-C_18:0_ acids is still considered as a potential paleotemperature proxy^24^; and was successfully applied to the reconstruction of paleotemperature in the surface water of Lake Biwa, Japan and in Guchenghu Lake, China^14^,^25^. The abrupt change in C_18:2_/C_18:0_ ratio at Yuchisi, over the course of a few decades, suggests that a rapid temperature change occurred at around 4,400 yr BP, when the Dawenkou Culture was replaced by the Longshan Culture. In addition, the alkane ratio, 2C_31_/(C_27_ + C_29_), exhibits abrupt changes around 4,400 yr BP (Fig. 2). The interpretation of this ratio is debated, but there is a consensus that it reflects climatic and environmental factors^24^,^25^. The dominance of longer chain lengths (C_31_, C_33_) could be a direct result of the impact of local climatic factors, such as aridity^24^, and therefore the increase in the ratio within YC1 may reflect the aridification of the local environment after 4,400 yr BP.

Within the YC1 profile, TOC and the Chenopodiaceae/total pollen ratio remain relatively stable during the Late Dawenkou Culture and the Longshan Culture. However, during the cultural transition layers, no pollen grains could be recovered, suggesting the occurrence of an extremely dry climatic event^21^. Although the total pollen did not vary significantly before and after the climatic transition at around 4,400 yr BP, the Chenopodiaceae/total pollen ratio increased from 2% to 22%. This finding is consistent with an increase in the alkane ratio 2C_31_/(C_27_ + C_29_) and indicates the occurrence of arid climatic conditions in the area after 4,400 BP, which is in agreement with the findings of our previous study at Yuchisi^21^.

The YC1 profile reveals abrupt changes in the concentrations of *n*-alkanols at around 4,400 yr BP. A significant change in *n*-C_18_-ol occurs in the layer corresponding to the transition between the Dawenkou and Longshan cultures; however, *n*-C_30_-ol exhibits only a small amount of variation. The concentrations of *n*-C_18_-ol and n-C_30_-ol exhibit respective increases from 0.68–4.78 μg/g, and from 0.28–0.47 μg/g, between 214 cm and 210 cm; followed by respective decreases to 0.83 μg/g and 0.19 μg/g at 206 cm. In general, the epicuticular waxes of higher plants contain even carbon number *n*-alkanols from C_26_ to C_30_, while lower plants (e.g., aquatic algae) have *n*-alkanol distributions dominated by C_16_ to C_22_ components^24^. The profiles of *n*-alkanols in profile YC1 suggest that the lower plants underwent more dramatic changes than did the higher plants.

## Discussion

### Time lag between climate changes and cultural responses

Several studies have pointed out that the chronologies of palaeoclimatic records and of archaeological material from different sites, even those in close proximity, could result in an apparent time lag between the climatic change and the vegetation response of up to 300 years^16^. The climatic impact of the “Holocene IRD Event 3”^26^ may have commenced as early as 4,500 yr BP, although the Neolithic cultural transformations occurred at about 4,200–4,000 yr BP, a lag of several hundred years^27^. In Egypt, the mid- to late Holocene climatic transition also occurred at around 4,500 calendar yr BP, several hundred years earlier than the collapse of Egyptian civilization at around 4,000 yr BP^28^. A similar observation has been made in the case of the Middle East^10^,^11^.

The length of the time lag depends upon the amplitude, abruptness, and duration of the climate change, and the stability of human societies. A less abrupt or lower amplitude change is more likely to enable the adaptation of both ecosystems and economies, and is thus may be less disruptive. In contrast, changes can be more devastating when they are abrupt and unpredictable^29^. For example, the climate changes associated with the Holocene IRD Event 3 appear to have been gradual in some regions, which allowed sufficient time for social and ecological systems to adapt^30^. However, the changes in paleotemperature biomarkers and the cultural transition at Yuchisi took place abruptly and simultaneously, and the human societal response was direct, with no time lag. This suggests the possibility that the Holocene IRD Event 3 may have been sufficiently intense in central China to have induced an almost immediate cultural response.

### Global linkage between abrupt climate changes and cultural collapses at around 4,400 yr BP

The period from 4,400–4,000 yr BP was important for both cultural evolution and climate change in China. In China, other fiefdom-like cultures, such as the Liangzhu, Shijiahe, Qijia, and Laohushan, had the potential to evolve into more complex societies, but they all collapsed from about 4,400–4,200 yr BP^27^. On a global scale, many notable prehistoric cultures such as the Akkadian empire of Mesopotamia; the pyramid-constructing Old Kingdom civilization of Egypt; the Harappan 3B civilization of the Indus valley; and the Early Bronze III civilizations of Palestine, Greece, and Crete; all reached their economic peaks around 4,300 yr BP. They then terminated abruptly before 4,200 yr BP as a result of catastrophic drought and cooling^10^,^11^,^31^,^32^,^33^. Furthermore, at around 4,500 yr BP, Paleoindians abandoned their campsites as the result of the drying out of lakes in the arid interior of Chile^34^, although their settlements persisted in the humid region of Peru^35^. In North America, Paleoindians left the High Plains and moved to the foot slopes of mountains due to the hot and dry climate^36^; and in East Africa, people migrated to cooler upland regions during this period^37^.

Numerous (over one hundred) natural archives from around the world record a remarkable climatic shift involving the sharply increased occurrence of drought, wetter conditions or floods, major dust storms, and possibly other climatic anomalies, at this time. The distribution of some of these events is illustrated in Fig. 3 11,27,32,38,39,40,41,42,43,44 (The locations are listed in Supplemental Table 1). This global event, which started around 4,400 yr BP and ended some 600 years later, has been variously termed the “4000 yr BP Event”^43^,^45^, the “4.2 ka event”^33^,^39^, and the “Bond Holocene IRD Event 3”^26^. It was the most significant cold period since the 8.2 ka event, and signified the climatic transition from the early Holocene warm period to the late Holocene, being characterized by minor climatic optimums which were then followed by a cold period^13^,^45^.

In China, records from mountain glaciers and lacustrine sediments document the occurrence of drought in the north and flooding in the south at around the same time, a situation linked theoretically with the collapse of prehistoric cultures^27^. Proxy climate records indicate wetter and/or cooler conditions in various parts of northern Europe and severe drought in southern Europe, as well as in western Asia, at around 4.2 ka^38^,^43^,^45^,^46^,^47^,^48^,^49^. In North America during the interval from 4300–4100 yr BP, a pronounced drought, with large and widespread ecological effects, impacted the region from the Great Lakes to the Central Plains, while the largest floods occurred in Arizona and Utah^39^,^41^. In Africa, more than twenty swamp and lake catchments record a shift to drier environmental conditions, while in South America a shift to a wetter environment occurred^43^. In summary, the climatic transition at 4,400 BP caused widespread anomalies including severe droughts, floods, dune reactivation, forest fires, and long-term changes in forest composition, with devastating societal and ecological effects^11^,^27^,^32^,^39^,^40^,^50^.

Numerous studies have considered the mechanism behind these climatic changes, including the role of the triggering of changes in the thermo- and hydrodynamic- circulation of the Earth caused by changes in solar irradiation, ocean variability, changes in atmospheric composition, and/or environmental threshold events^38^,^42^. Solar irradiation is the major energy input to the Earth’s climate system, and increases in solar irradiation enhance easterly winds, leading to poleward shifts in the positions of the subtropical westerly jets, broadening of the tropical Hadley circulations, and poleward shifts in storm tracks, and vice versa^51^,^52^,^53^. Bond and others documented a similar cold event (at 4.2 ka) in the North Atlantic, and noted a corresponding increase in cosmogenic isotopes, suggesting a reduction in solar irradiation at this time^54^. Thus, with the reduced solar irradiation, many regions in the world experienced a significant change in climatic conditions, and the global climate system appears to have been unstable during this transition.

The causes of prehistoric cultural collapse have been long debated. Generally, archaeologists and sociologists have attributed such collapses to complex social, political, economic, and environmental factors^1^. Nomadism and agriculture, or nomadic hunting and gathering activities, were the main means of human subsistence prior to the Industrial Revolution. Climatic conditions such as temperature, precipitation and water availability had profound impacts on food, water and energy supplies and other resources vital for prehistoric arable and pastoral societies^33^,^55^. Disruption of these resources would have caused famine, disease, death, social upheaval, and the collapse of pre-modern societies^2^. Recently, more attention has been paid to the impact of severe climate changes on pre-modern civilizations; in a significant number of cases, major societal changes coincided with major climatic anomalies^45^. This dynamic is also likely to have been applicable to prehistoric cultures: for example, changes in climatic conditions have also been proposed as an important factor in the collapse of the Dawenkou Culture, in addition to overpopulation, famine, and wars^27^,^56^.

War, drought, and famine have impacted local cultures throughout the ages; however, there are disparities in the timing of the collapses of isolated cultures in different geographical areas. The collapse of diverse and widely-distributed civilizations at around the same time cannot be adequately explained in terms of general economic and social factors and it may be more reasonable to regard the global climatic transition at around 4,400 BP as being responsible for precipitating these well-documented widespread cultural collapses.

## Methods

### Environmental context and sampling

**Figure 1:**
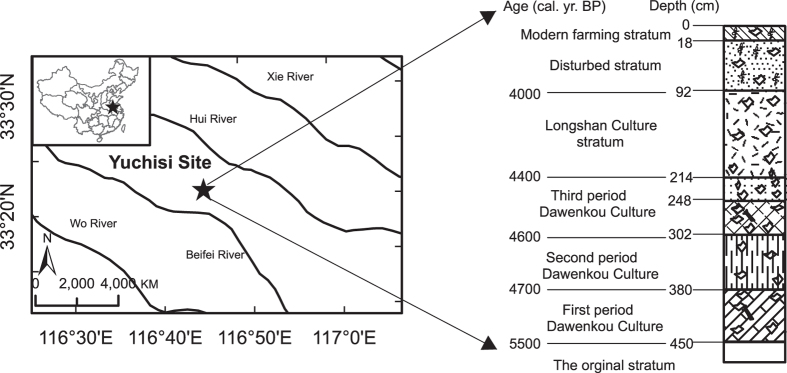
Left: Location of the Yuchisi site and YC1 profile in relation to local river systems (inset map shows the location of the study area in China). Right: Stratigraphy, chronology, and cultural context of the studied sedimentary deposit^21^. The map was created with ArcGIS 10.3.1 (http://www.esri.com/software/arcgis).

**Figure 2:**
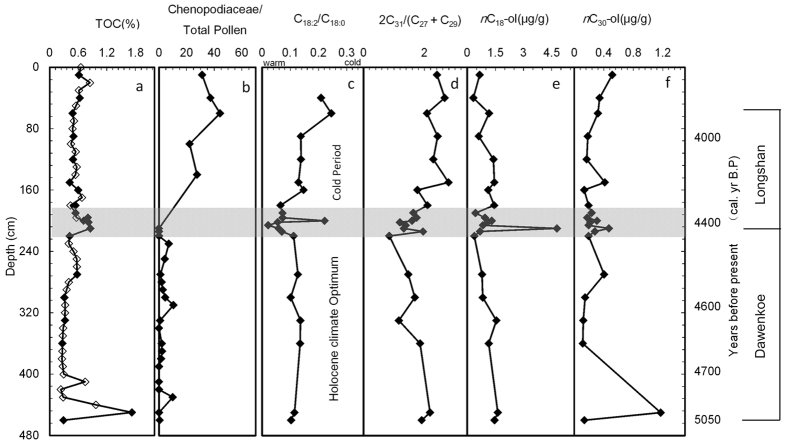
Profiles of biomarkers and other environmental proxies in the YC1 sediment profile. (**a**) TOC; (**b)** the ratio of Chenopodiaceae to total pollen^21^; (**c**) the acid ratio of C_18:2_/C_18:0_; (**d)** the alkanes ratio of 2C_31_/(C_27_ + C_29_); (**e**) *n*C_18_-ol; (**f**) *n*C_30_-ol.

**Figure 3:**
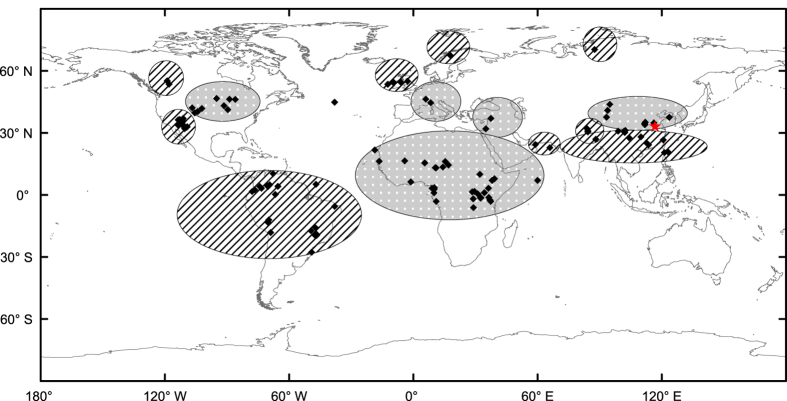
Global distribution of the “Bond Holocene IRD Event 3”. The red star represents the Yuchisi site. The hatched areas were affected by drought or dust storms, and the dotted areas by wet conditions or flooding. See text for references. The map was created with ArcGIS 10.3.1 (http://www.esri.com/software/arcgis).

The Yuchisi archeological site is located between the Yellow and Huai Rivers, in Mengcheng County (32°55′-33°29′N, 116°16′-116°46′E), Anhui Province, eastern China (Fig. 1). It was excavated by the Archeological Institute of the Chinese Academy of Social Sciences (AI-CASS). With an area about 100,000 m^2^, it is the largest and most integrated site with abundant prehistoric architectural remains in eastern China. A number of building foundations, with room numbers from 1 to 5, have been excavated. Since 1990, archeologists from AI-CASS have explored the site 13 times and discovered numerous bones, porcelain shards, and mortuary objects. The Yuchisi site is exposed roughly at the center of a hill and 2–3 m above ground; it is bounded on its western and southern sides by small unsurfaced roads, and on its eastern side by a modern artificial canal. The hill approximates a square with dimensions of 370 m (east-west) by 250 m (north-south). The remains of an ancient moat, which surrounds the architectural complex of the late Dawenkou Culture, are evident at the site. The moat has a north-south span of 230~240 m, an east-west span of 220 m, a width of 25–30 m, and a depth of 4.5 m. The moat forms a circle with an outlet in the south-west corner. Along the north-south axis of the moat and in its center are three archaeological excavation pits.

We sampled a sediment profile, named YC1, from the south wall of the central excavation pit above the moat water level. This pit was 4.6-m-deep, and its cultural layers and its chronology have been studied in detail; 230 samples were collected from the south wall at an interval of 2 cm. From top to bottom, the pit was divided into eight layers or strata (Fig. 1), and the boundaries of each stratum were assigned in the field under the direction of Prof. Wang Jihuai from AICASS.

Chronological control is based on the cultural boundary depths determined by AI-CASS^17^,^18^. Charcoal fragments from the profile were dated at the Key Laboratory of Heavy Ion Physics, Peking University, in the Accelerator Mass Spectrometry (AMS) Facility. The correlation coefficient between age and depth is 0.986 (p < 0.01), and the average sediment accumulation rate is close to 0.38 cm/yr. There are no age reversals and no obvious change in the accumulation rates of different layers, indicating the absence of significant disturbance occurred during the deposition history of YC1 (see detailed information in^21^).

TOC and inorganic elements were analyzed every 10 cm, and the pollen assemblages of selected layers were analyzed and published previously^21^. In the present study, we analyzed 16 samples from YC1 (5,050–4,000 yr BP). The sample depths were selected based upon previous TOC analyses and the results of archaeological exploration^21^.

### Analysis of biomarkers

The sediment samples were collected and stored in pre-cleaned brown glass jars. For organic biomarker analysis, freeze-dried sediment samples were Soxhlet extracted for 72 h with a 2:1 dichloromethane/methanol mix. The extracts were concentrated by rotary evaporation and then saponified using 0.5 M KOH/MeOH. Neutral lipids were partitioned out of the basic solution with hexane. The pH of the saponified extract was then brought to 2 using 6 N HCl, and acidic lipids were extracted with 20% methylene chloride in hexane. The acidic lipids were kept in anhydrous Na_2_SO_4_ overnight to remove ant traces of water. The neutral lipids were further separated using (5% deactivated) silica gel column chromatography using solvents of increasing polarity from hexane to methylene chloride. The alcohol and acid fractions were treated with *N,O*-bistrimethylsilyltrifluoroacetamide (BSTFA) to form trimethylsilyl (TMS)-ether and ester derivatives.

The alkanes and the derivatives of the alcohol and acidic fraction were analyzed using an HP 5890 gas chromatograph-mass spectrometer with a DB-5 ms (50 m × 0.32 mm and 0.25 μm film thickness) capillary column (J&W). Helium was used as the carrier gas. The mass spectrometer was operated in EI mode at 70 eV. The MS data was acquired in the full mode and processed with the Chemstation data system.

The GC oven for alkanes, alcohols and acid fractions was programmed as follows. Alkanes: hold 2 min at 60 °C, increase to 200 °C at 7 °C/min, and then increase to 280 °C at 3 °C/min, and hold 30 min at 280 °C. Alcohols: hold 2 min at 60 °C, increase to 200 °C at 10 °C/min, increase to 280 °C at 3.5 °C/min, hold 15 min at 280°C, increase to 300 °C at 1.5 °C/min, and hold 30 min at 300 °C. Acids: hold 2 min at 60 °C, increase to 150 °C at 10 °C/min, increase to 300 °C at 2.5 °C/min, hold 30 min.

The recovery results of the n-alkanes, alcohols, and acids in our experiment were 95.8 ± 2.9%, 92.3 ± 2.3% and 93.7 ± 1.6%, respectively.

## Additional Information

**How to cite this article**: Wang, J. *et al*. The abrupt climate change near 4,400 yr BP on the cultural transition in Yuchisi, China and its global linkage. *Sci. Rep.* **6**, 27723; doi: 10.1038/srep27723 (2016).

## Supplementary Material

Supplementary Information
