# Supplementary material for: The abrupt climate change near 4,400 yr BP on the cultural transition in Yuchisi, China and its global linkage
Source: Sci Rep. 2016 Jun 10;6:27723. doi: 10.1038/srep27723 (PMC4901284; doi:10.1038/srep27723)
Supplement: Supplementary Information [file srep27723-s1.pdf]

The abrupt climate change near 4,400 yr BP on the cultural transition in Yuchisi, China and its global linkage

Jianjun Wang[1,2], Liguang Sun[2], Liqi Chen[1], Libin Xu[2,3], Yuhong Wang[4], Xinming Wang[5]

[1] Key Laboratory of Global Change and Marine-Atmospheric Chemistry, Third Institute of Oceanography, State Oceanic Administration, Xiamen, China

[2] Institute of Polar Environment, University of Science and Technology of China, Hefei, China

[3] Zhangjiagang Water Supply & Drainage Company, Zhangjiagang, China

[4] National Institute of Health, USA

[5] Guangzhou Institute of Geochemistry, Chinese Academy of Sciences, Guangzhou, China

Correspondence and requests for materials should be addressed to J.W. (email: wangjianjun@tio.org.cn) or L.S. (email: slg@ustc.edu.cn)

Supplemental Table1 A partial location list of "Bond's Holocene IRD Event 3" in Figure 3

Location	Lon	Lat	Climate Change	References
Yuchisi, China	116.520	32.910		this study
	94.943	43.824		
	93.861	40.911		
	93.316	37.629		
	83.921	32.101		
	84.341	31.001		
	84.279	30.514		
	88.132	26.989		
North China	98.921	30.883	Drought	Wu and Liu, 2004 and refs therein
	102.408	31.182		Xia et al. 2003
	101.746	30.758		
	102.112	30.216		
	104.222	27.664		
	123.612	37.566		
	111.990	35.194		
	111.687	34.527		
	111.866	33.985		
	116.147	34.896		
	109.943	28.088		
	120.724	26.627		
South China	113.072	24.743	Flood	Wu and Liu, 2004 and refs therein
	113.617	24.256		
	122.896	20.911		
	121.269	20.487		
Altantic	-38.011	44.875	Cold	Bond et al. 2001
	-12.351	53.524		
	-9.503	54.440		
England	-2.770	55.000	Wet	Barber et al. 2003
	-9.500	54.200		Hughes et al. 2000
	-6.240	54.710		
	59.040	24.400	Dust	Cullen et al. 2000
	6.000	46.370		
	8.373	44.577	Drought	Drysdale et al. 2006
Europe	35.000	32.000		
	37.446	37.024		
	22.830	68.680		Korhola et al. 2000
	18.070	67.360	Wet	Perry and Hsu 2000

	87.550	70.370		Laing and Smol 2003
	-106.807	42.149		
	-104.188	40.335		
	-102.156	41.817		
	-95.052	46.663		
North America	-91.458	43.178	Drought	Booth et al. 2005 and refs therein
	-89.641	41.267		
	-88.797	46.307		
	-85.964	46.257		
	-105.600	39.730		
	32.500	-1.500		
	29.050	-6.140		
	28.930	-1.850		
	31.100	0.500		
	29.540	1.700		
	32.480	-1.170		
	34.300	1.100		
	36.450	-1.200		
	36.940	-2.400		
	38.050	7.030		
	39.140	7.850		
	36.250	3.250		
	28.450	1.500		
	10.150	3.560		
Africa	10.080	2.850	Drought	Marchanta and Hooghiemstra 2004, and refs therein
	-1.250	6.300		
	9.050	3.240		
	17.000	14.500		
	14.300	13.500		
	-16.500	16.300		
	37.350	-3.080		
	-18.600	21.750		
	10.000	1.000		
	15.400	16.200		
	5.500	15.500		
	11.010	13.120		
	10.560	13.180		
	-4.150	16.500		
	32.100	10.010		
	11.040	-3.140		
	-110.434	32.126		

	-110.329	32.484		
	-109.414	32.790		
	-109.212	32.990		
	-110.795	33.400		
	-111.124	33.853		
	-111.654	33.969		
	-111.799	34.411		
North America	-113.253	34.273	flood	Ely et al. 1993
	-113.566	34.326		
	-113.831	33.947		
	-111.485	35.390		
	-112.482	36.094		
	-111.631	36.283		
	-111.766	36.389		
	-111.197	36.862		
	-111.188	37.031		
	-112.907	36.547		
	-113.598	36.315		
	-118.900	55.200		
	-118.000	53.600		

	-68.880	-18.160		
	-70.050	-13.450		
	-69.850	-12.080		
	-66.680	0.260		
	-46.680	-19.500		
	-49.450	-17.280		
	-47.580	-15.560		
	-47.450	-19.560		
	-47.030	5.180		
	-46.780	-19.000		
South America	-38.000	-5.500	Wetter	Marchanta and Hooghiemstra
	-49.150	-27.700		2004,and refs therein
	-77.890	1.660		
	-76.600	2.530		
	-70.210	4.050		
	-74.140	4.040		
	-73.540	3.460		
	-73.350	3.220		
	-70.540	4.450		
	-69.450	4.950		
	-65.450	4.150		
	-67.750	10.320		

References

Barber KE, Chambers FM, Maddy D. Holocene palaeoclimates from peat stratigraphy: macrofossil proxy climate records from three oceanic raised bogs in England and Ireland. *Quat. Sci. Rev.* **22**, 521-539 (2003).

Bond G*, et al.* Persistent Solar Influence on North Atlantic Climate During the Holocene. *Science* **294**, 2130 (2001).

Booth RK*, et al.* A severe centennial-scale drought in midcontinental North America 4200 years ago and apparent global linkages. *The Holocene* **15**, 321-328 (2005).

Cullen HM*, et al.* Climate change and the collapse of the Akkadian empire: Evidence from the deep sea. *Gelology* **28**, 379-382 (2000).

Drysdale R*, et al.* Late Holocene drought responsible for the collapse of Old World civilizations is recorded in an Italian cave flowstone. *Geology* **34**, 101-104 (2006).

Ely LL, Enzel Y, Baker VR, Cayan DR. A 5000-Year Record of Extreme Floods and Climate Change in the Southwestern United States. *Science* **262**, 410-412 (1993).

Hughes PDM, Mauquoy D, Barber KE, Langdon PG. Mire-development pathways and palaeoclimatic records from a full Holocene peat archive at Walton Moss, Cumbria, England. *Holocene* **10**, 465-475 (2000).

Korhola A, Weckstrom J, Holmstrom L, Erasto P. A Quantitative Holocene Climatic Record from Diatoms in Northern Fennoscandia. *Quat. Res.* **54**, 284-294 (2000).

Laing TE, Smol JP. Late Holocene environmental changes inferred from diatoms in a lake on the western Taimyr Peninsula, northern Russia. *J. Paleolimnol.* **30**, 231-

247 (2003).

Marchant R, Hooghiemstra H. Rapid environmental change in African and South American tropics around 4000 years before present: a review. *Earth Sci. Rev.* **66**, 217-260 (2004).

Perry CA, Hsu KJ. Geophysical, archaeological, and historical evidence support a solar-output model for climate change. *Proc. Natl. Acad. Sci. U. S. A.* **97**, 12433-12438 (2000).

Xia Z, Yang X, Ye M. Prehistorical hazards of Lajia Site, Qinghai, China. *Chin. Sci. Bull.* **48**, 1200-1204 (in Chinese) (2003).
